# Tree growth responses to temporal variation in rainfall differ across a continental-scale climatic gradient

**DOI:** 10.1371/journal.pone.0249959

**Published:** 2021-05-04

**Authors:** Alison J. O’Donnell, Michael Renton, Kathryn J. Allen, Pauline F. Grierson

**Affiliations:** 1 School of Biological Sciences, The University of Western Australia, Perth, Western Australia, Australia; 2 School of Agriculture and Environment, The University of Western Australia, Perth, Western Australia, Australia; 3 School of Ecosystem and Forest Sciences, The University of Melbourne, Richmond, Victoria, Australia; 4 ARC Centre of Excellence in Australian Biodiversity and Heritage, University of New South Wales, Sydney, New South Wales, Australia; Universiti Teknologi Malaysia, MALAYSIA

## Abstract

Globally, many biomes are being impacted by significant shifts in total annual rainfall as well as increasing variability of rainfall within and among years. Such changes can have potentially large impacts on plant productivity and growth, but remain largely unknown, particularly for much of the Southern Hemisphere. We investigate how growth of the widespread conifer, *Callitris columellaris* varied with inter-annual variation in the amount, intensity and frequency of rainfall events over the last century and between semi-arid (<500 mm mean annual rainfall) and tropical (>800 mm mean annual rainfall) biomes in Australia. We used linear and polynomial regression models to investigate the strength and shape of the relationships between growth (ring width) and rainfall. At semi-arid sites, growth was strongly and linearly related to rainfall amount, regardless of differences in the seasonality and intensity of rainfall. The linear shape of the relationship indicates that predicted future declines in mean rainfall will have proportional negative impacts on long-term tree growth in semi-arid biomes. In contrast, growth in the tropics showed a weak and asymmetrical (‘concave-down’) response to rainfall amount, where growth was less responsive to changes in rainfall amount at the higher end of the rainfall range (>1250 mm annual rainfall) than at the lower end (<1000 mm annual rainfall). The asymmetric relationship indicates that long-term growth rates of *Callitris* in the tropics are more sensitive to increased inter-annual variability of rainfall than to changes in the mean amount of rainfall. Our findings are consistent with observations that the responses of vegetation to changes in the mean or variability of rainfall differ between mesic and semi-arid biomes. These results highlight how contrasting growth responses of a widespread species across a hydroclimatic gradient can inform understanding of potential sensitivity of different biomes to climatic variability and change.

## Introduction

Spatial patterns of average primary productivity and carbon fluxes among biomes can be strongly predicted by spatial variation in mean annual rainfall [1–4]. However, the temporal dynamics of productivity in relation to inter-annual variability of rainfall are more poorly resolved [2, 4–7]. In particular, how vegetated ecosystems will respond to observed and projected shifts in the mean amount and/or variability of rainfall, as well as the seasonal timing, frequency, and intensity of rainfall events (e.g., [8–12]) remains uncertain. Understanding how temporal patterns of rainfall influence plant productivity and growth is thus crucial for modelling and predicting vegetation responses under present or future climate conditions.

The sensitivity of plant productivity to changes in the mean or variability of rainfall depends on the slope and shape (linear or non-linear) of the relationship between them [5–7, 13, 14]. Temporal relationships between productivity and rainfall are typically portrayed as linear across a range of biomes [14, 15]. Consequently, it is also often assumed that a change in the mean amount of rainfall will result in a proportional change in mean productivity, while a change in rainfall variance will not significantly affect mean productivity [6, 7, 13]. However, recent evidence suggests that relationships between productivity and rainfall are often non-linear [15]. Non-linear (‘concave-up’ or ‘concave-down’) relationships between productivity and rainfall indicate that extremes of annual or seasonal rainfall, either positive or negative, can potentially drive disproportionately large (asymmetrical) responses in plant productivity [6, 7, 13]. For example, if the relationship is of a concave down form, dry extremes will drive larger decreases in productivity relative to increases in productivity driven by wet extremes (‘negative asymmetry’), and thus increases in rainfall variability are expected to drive both increased variability in productivity and an overall decrease in mean productivity [6, 16, 17].

The shape of the relationship between rainfall and productivity tends to differ between water-limited and mesic biomes (i.e., the ‘S’ curve concept; [1, 13]). In water-limited (i.e., arid and semi-arid) biomes, the relationship between rainfall and productivity is typically either linear or of a ‘concave-up’ (positive asymmetry) non-linear form, where wet years result in increases in productivity and dry years result in decreases in productivity of either a proportional magnitude if linear, or a smaller magnitude if concave up (e.g., [2, 18]). In contrast, in mesic biomes, productivity can show a ‘concave-down’ (negative asymmetry) response to rainfall amount, reaching a maximum or even declining with increasing water availability as other factors such as light or nutrient availability become more limiting (e.g., [5, 19]). Despite evidence for non-linear reponses of productivity to rainfall amount in some biomes, how common non-linear relationships are within and among biomes or how well supported they are over linear models is still poorly quantified [5, 15, 18].

Much of what is known about the temporal relationship between rainfall and productivity is based on studies that have monitored responses of above-ground primary productivity to water availability under either natural or manipulated rainfall regimes, but these are generally over relatively short time frames (i.e., typically <15 years; see e.g., [2, 4, 5, 20, 21]) and rarely include extreme rainfall conditions [18, 19]. Knapp et al. [15] showed that empirical support for non-linear, particularly concave-down relationships between productivity and rainfall is rare because extreme rainfall years are poorly represented in many productivity datasets. In addition, Knapp et al. [15] indicate that relationships between productivity and rainfall can change between linear and non-linear forms depending on whether rainfall extremes are included. For example, a recent study by Dannenberg et al. [17] analysed more than 100 years of tree ring and rainfall data, including observations of both extreme wet and extreme dry years and demonstrated that non-linear (concave-down) relationships between tree growth and rainfall are common in the semi-arid west of the United States. However, such multi-decadal studies are rare. Consequently, many of the current temporal models of productivity-rainfall relationships that are based on short-term datasets may not be appropriate for predicting responses to changes in rainfall extremes [15, 18].

Here, we use tree-ring records and high-quality instrumental rainfall data that span more than 110 years and include both extreme dry and extreme wet conditions to investigate the shape and strength of temporal relationships between rainfall and tree growth in Australia. We use a spatial network of five tree-ring chronologies encompassing a broad climatic and latitudinal (12–33°S) range across northern and western Australia to also investigate how temporal relationships between growth and rainfall vary between mesic (tropical) and water-limited (semi-arid) biomes. While these are relatively few sites compared to other continents, the western half of Australia is a remarkable area in the world to develop a tree-ring network across a large latitudinal range as this region is without significant changes in topography and shows strong spatial coherence in rainfall patterns [22, 23]. Consequently, relatively few chronologies are needed to capture spatial patterns in climate [23].

Our tree-ring records are based on ring widths of the widespread Australian conifer, *Callitris columellaris* F.Muell., one of the few Australian tree species that produces clear growth rings. *C*. *columellaris* exhibits hydraulic traits (shallow roots, anisohydric stomatal control and a highly opportunistic water use strategy; [24]) that are also observed in other Australian small tree species, including the *Acacia aneura* Benth. species complex, which dominate much of the semi-arid and arid woodlands of Australia [25, 26]. Consequently, we use *C*. *columellaris* as an indicator species of the likely response of shallow-rooted woody vegetation more broadly to inter-annual variation in rainfall.

Our first aim was to quantify the slope and shape of the temporal relationship between annual tree growth and rainfall amount and determine if these relationships differ between semi-arid and mesic biomes. Based on previous studies of plant productivity responses to inter-annual variation in rainfall, we expect that the relationship between tree growth and rainfall in semi-arid biomes will be strong and of either a linear or ‘concave-up’ form, whereas in the mesic (tropical) biomes, we expect the relationship to be weaker and of a ‘concave down’ form. The relationship between rainfall amount and tree growth may also differ widely among biomes with similar mean annual rainfall depending on the seasonal distribution of rainfall. In particular the seasonal timing, duration, frequency, and intensity of rain events may be as important for driving growth as the amount of rainfall that is delivered [17, 27, 28]. Our second aim was thus to determine whether other attributes of the rainfall distribution (i.e., frequency, intensity, intermittency and seasonality), which may differ both within and among biomes, are potentially also important drivers of growth.

## Site descriptions

### Ecology and taxonomy of *Callitris columellaris*

Plant productivity and growth is primarily limited by water availability across the vast majority of the Australian continent [29, 30]. Accordingly, rainfall amount is a strong driver of patterns in the secondary growth of our target species, *Callitris columellaris* across its range (e.g., [31–34]). *C*. *columellaris* is widespread across Australia and has a broad climatic range, inhabiting some of the driest regions of Australia, including the arid (< 250 mm annual rainfall) and semi-arid interior (<500 mm annual rainfall) as well as higher rainfall areas, including the tropical north of the continent where mean annual rainfall can exceed 1,200 mm [35–37].

All of the trees used in this study are the single species *C*. *columellaris*, which includes *C*. *intratropica* R.T.Baker & H.G.Sm. and *C*. *glaucophylla* Joy Thomps. & L.A.S.Johnson as taxonomic synonyms of *C*. *columellaris* following the convention of Farjon [38] and the Western Australian Herbarium (https://florabase.dpaw.wa.gov.au/browse/profile/8466). Samples were collected with permission from the Western Australian Department of Biodiversity, Conservation and Attractions under Flora Collection License SW019556 issued to A. O’Donnell.

### Climate zones

**Fig 1:**
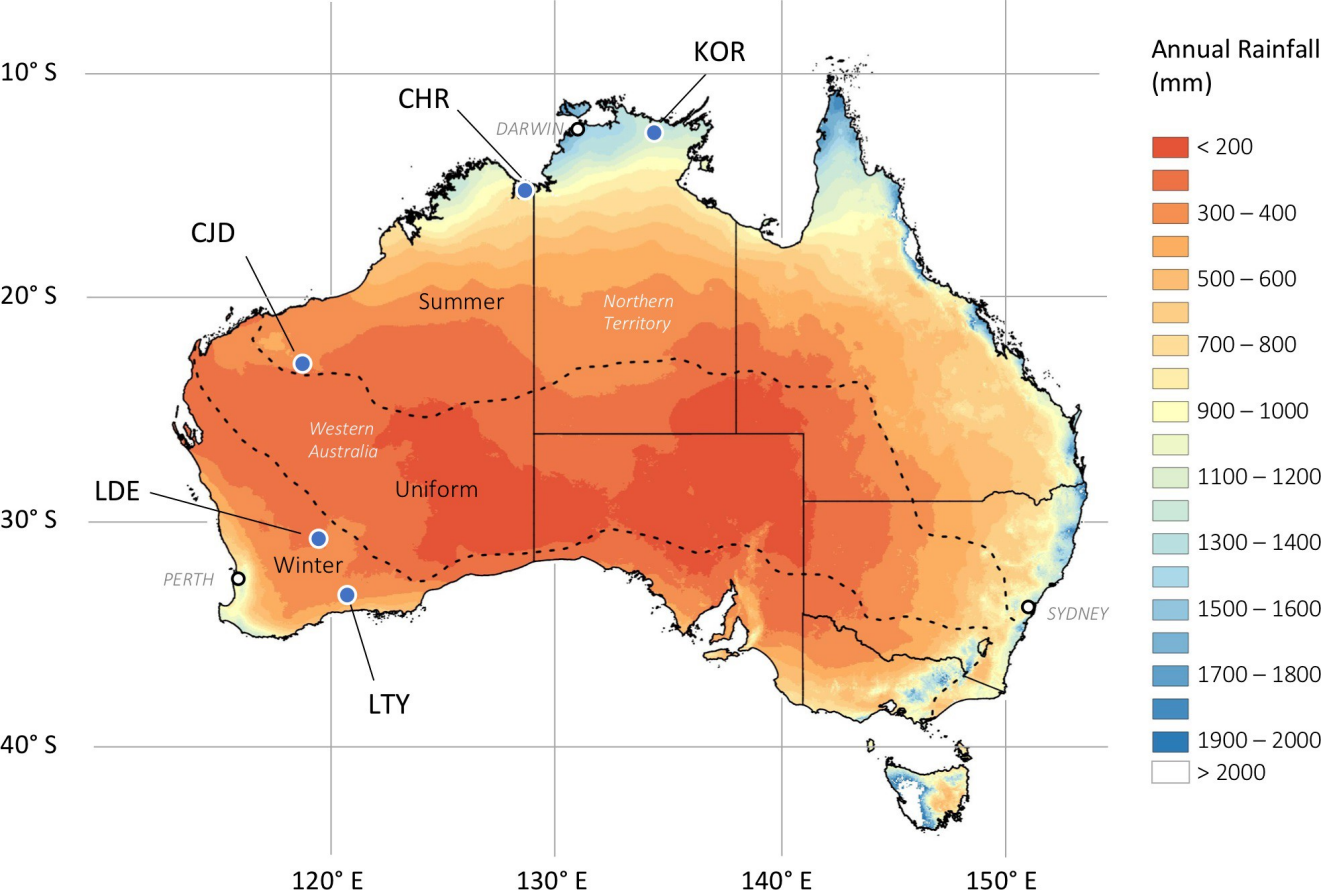
Location of five *Callitris columellaris* tree-ring sites in relation to annual rainfall and rainfall seasonality zones of Australia. Coloured shading indicates the 1961–1990 mean annual (January to December) rainfall amount (Data sourced from the Australian Bureau of Meteorology: http://www.bom.gov.au/jsp/ncc/climate_averages/rainfall/index.jsp). Dashed lines indicate boundaries between areas with summer-dominated, uniform (both summer and winter) or winter-dominated rainfall distributions (Data sourced from the Australian Bureau of Meteorology: http://www.bom.gov.au/jsp/ncc/climate_averages/climate-classifications/index.jsp?maptype=seasb#maps). Solid lines indicate state and territory boundaries and white dots indicate the location of some state capital cities.

Our study includes *C*. *columellaris* trees from five sites spanning much of the latitudinal gradient of mainland Australia and encompassing three distinct climate zones (Fig 1; description below). Water availability varies dramatically between the northern tropical zone where annual rainfall is high, reliable and falls in a strongly seasonal pattern and the arid/semi-arid zone where annual rainfall is low, unreliable, and less restricted to specific seasons (Figs 1 and S1).

#### Tropical

The two northernmost sites, Korlobirrahda (KOR) and Carlton Hill (CHR) are located in the monsoonal tropical zone of northern Australia (Fig 1). The northernmost site, KOR, is located on the Arnhem Plateau (12.55°S, 134.37°E) in the Northern Territory. The CHR site is located on Carlton Hill Station (15.09°S, 128.68°E), a pastoral lease in the east Kimberley Region of Western Australia. The climate of both tropical sites is characterised by hot, wet summers (Dec-Mar) with distinct dry seasons in the Austral winter-spring months (Jun-Sep; S1 Fig). Both sites are considered to be in ‘mesic’ climate zones, receiving high annual rainfall (>800 mm). However, CHR typically receives 400 mm less rainfall than KOR and experiences greater year-to-year variability in annual rainfall (CV = 34% vs 21%; Table 1). At both sites, *C*. *columellaris* trees form scattered to open savanna woodlands with a grassy understorey, generally on sandplains or low sandstone ridges.

**Table 1:** Summary of climate and rainfall data for each of the five *Callitris columellaris* sites and the annual and growing season periods used in further analyses.

Site	Climate	Rainfall Data Coverage	Annual Period	Mean Annual Rainfall (mm)	CV of Annual Rainfall	Growing Season	Length of growing season (months)
KOR	Wet Tropical	1912–2018	pSep-Aug	1,287	21%	Mar-Jun	4
CHR	Tropical	1908–2016	pJun-May	863	34%	pJun-May	12
CJD	Sub-tropical semi-arid	1908–2018	pDec-Nov	373	46%	pDec-May	6
LDE	Mediterranean semi-arid	1908–2018	pNov-Oct	305	28%	Feb-Aug	7
LTY	Mediterranean semi-arid	1908–2018	pNov-Oct	408	21%	Jan-Sep	9

CV is the coefficient of variation. Growing season is the period of consecutive months that showed the highest Pearson correlation between rainfall and tree growth (ring-width index). Lower case ‘p’ indicates the month is in the calendar year prior to the main growing season.

#### Sub-tropical semi-arid

The Juna Downs (CJD) site is located in the sub-tropical semi-arid Pilbara region of northwest Australia (22.83°S, 118.62°E; Fig 1). The climate here is characterised by hot summers and cool winters (S1 Fig). CJD receives ~375 mm of rainfall annually, which falls mainly in the Austral summer months (Dec-Mar; S1 Fig). Unlike the two tropical sites, summer rainfall at CJD is typically dominated by irregular and unpredictable extreme rainfall events (tropical cyclones and other closed lows) rather than monsoonal rainfall [39]. Consequently, CJD experiences greater year-to-year variability in annual rainfall than either the tropical sites or the southern semi-arid sites (CV of annual rainfall = 46%; Table 1). The CJD site may also receive low rainfall in the winter months (Apr-Jul), while the spring months (Aug-Oct) are typically dry (S1 Fig). At CJD, *C*. *columellaris* forms an open woodland restricted to a fire protected, south-facing, shallow gully on skeletal and rocky soils.

#### Mediterranean semi-arid

The southern sites, Lake Tay (LTY; 33.02°S, 120.74°E) and Lake Deborah (LDE; 30.68°S, 119.27°E), are both located in the Mediterranean semi-arid climate zone of southwest Australia (Fig 1). The climate here is characterised by hot, dry summers and cool, wet winters (S1 Fig). Mean annual rainfall is low at both sites, but LDE receives ~100 mm less annual rainfall than LTY (305 mm vs. 408 mm; Table 1). In contrast to the three northern sites, rainfall at LDE and LTY occurs mainly in the Austral autumn-winter months (Mar-Sep), but is more evenly distributed throughout the year and rarely exceeds 100 mm in a single month (S1 Fig). At both LDE and LTY, *C*. *columellaris* trees form sparse woodlands adjacent to large ancient saline lake systems.

While rainfall seasonality varies among the northern and southern sites, temperature seasonality does not. For all sites, the coldest months are in the Austral winter (Jun-Aug) and the warmest are in the Austral summer (Dec-Feb). However, the temperature range varies greatly among sites (S1 Fig). While minimum temperatures at the southern sites are occasionally less than 0°C in the wet season and therefore potentially limiting to xylogenesis [40–42], this occurs rarely and only for short periods (days). In addition, day time (maximum) temperatures during the wet season at all of the sites typically exceed 15°C (S1 Fig), suggesting that low temperatures are unlikely to be limiting to growth at any of the sites.

## Materials and methods

### Ring-width data

We used five chronologies of ring-width indices: three published (LTY, [34]; CJD, [33]; KOR, [31]) and two new ring-width chronologies (LDE and CHR). We used ring-width indices (RWI) instead of raw ring-width measurements because the width of growth rings typically decline as the age of trees and the girth of their trunks increases. RWI provides an index of the inter-annual variability of ring widths within a population of trees, but is standardised to a mean of one across all sites, so does not provide an indication of the potential differences in radial growth rates among sites with different climatic attributes. As we were primarily interested in the inter-annual variability of ring widths in relation to climatic variability we have used RWI for all further analyses but provide a summary of the raw ring width data (S2 Fig) for comparison of actual growth rates among our sites.

To account for potential age-related decline in ring width, we statistically detrended the raw ring-width measurements to remove age-related (non-climatic) trends and converted them to ring-width indices as residuals from the detrending curve [43]. The LTY, LDE, CJD and CHR ring-width series were first power-transformed to stabilise variance [43] and detrended using an age-dependent spline. The KOR ring-width chronology was used here as it was published in Allen et al. [31]. The KOR series were also power-transformed prior to detrending with a negative exponential or linear model. All series for all sites were detrended in a signal free environment [44] using the RCSigFree program (http://www.ldeo.columbia.edu/tree-ring-laboratory/resources/software). Further details on the sample size, detrending methods and statistical quality can be found in O’Donnell et al. [33] for the CJD chronology and Allen et al. [31] for the KOR chronology. Details of the previously published LTY chronology (1655–2005 CE) can be found in Cullen and Grierson [34]. However, we have now extended the LTY chronology to 2013 (see S3 Fig for details). Details of the sample size and statistical quality of the two new ring-width chronologies (LDE and CHR) can also be found in S3 Fig.

### Rainfall and temperature data

Our study sites are located in very remote areas; there are few weather stations and hence long, high-quality instrumental records within a 50 km radius of each site. Hence, we used the gridded SILO product [45] downloaded for the nearest 0.5° grid point to each site for daily minimum and maximum temperature data (available from: https://www.longpaddock.qld.gov.au/silo/). The estimated interpolation error of the SILO data has been thoroughly examined by Jeffrey et al. [45]. Interpolation error at the tropical sites (CHR and KOR) is higher than at the semi-arid sites (CJD, LTY and LDE) due to a lower density of instrumental weather stations around the tropical sites [45; their Figs 5 and 6]. The SILO daily rainfall data product shows generally low to moderate interpolation error across the five study sites [45; their Fig 17]; however, we found several large and unexplained inconsistencies between the SILO rainfall data and instrumental rainfall data from stations in the region of the Lake Tay (LTY) site in the 1950s and 1960s. For rainfall data, we instead used a 2° x 2° search area centred on site locations to find weather stations that each had long records (an arbitrarily chosen minimum of 60 years) of instrumental daily rainfall data (data available from Australian Bureau of Meteorology, http://www.bom.gov.au/climate/data/). We calculated the mean of daily rainfall data from the stations meeting these criteria for each site. The spatial coverage and number of available stations (rainfall records) varied by site–we found only three stations that met our criteria for the KOR site while the other sites had 6–8 records available (see S1 Table for details of station rainfall data).

### Annual and ‘growing season’ periods

**Fig 2:**
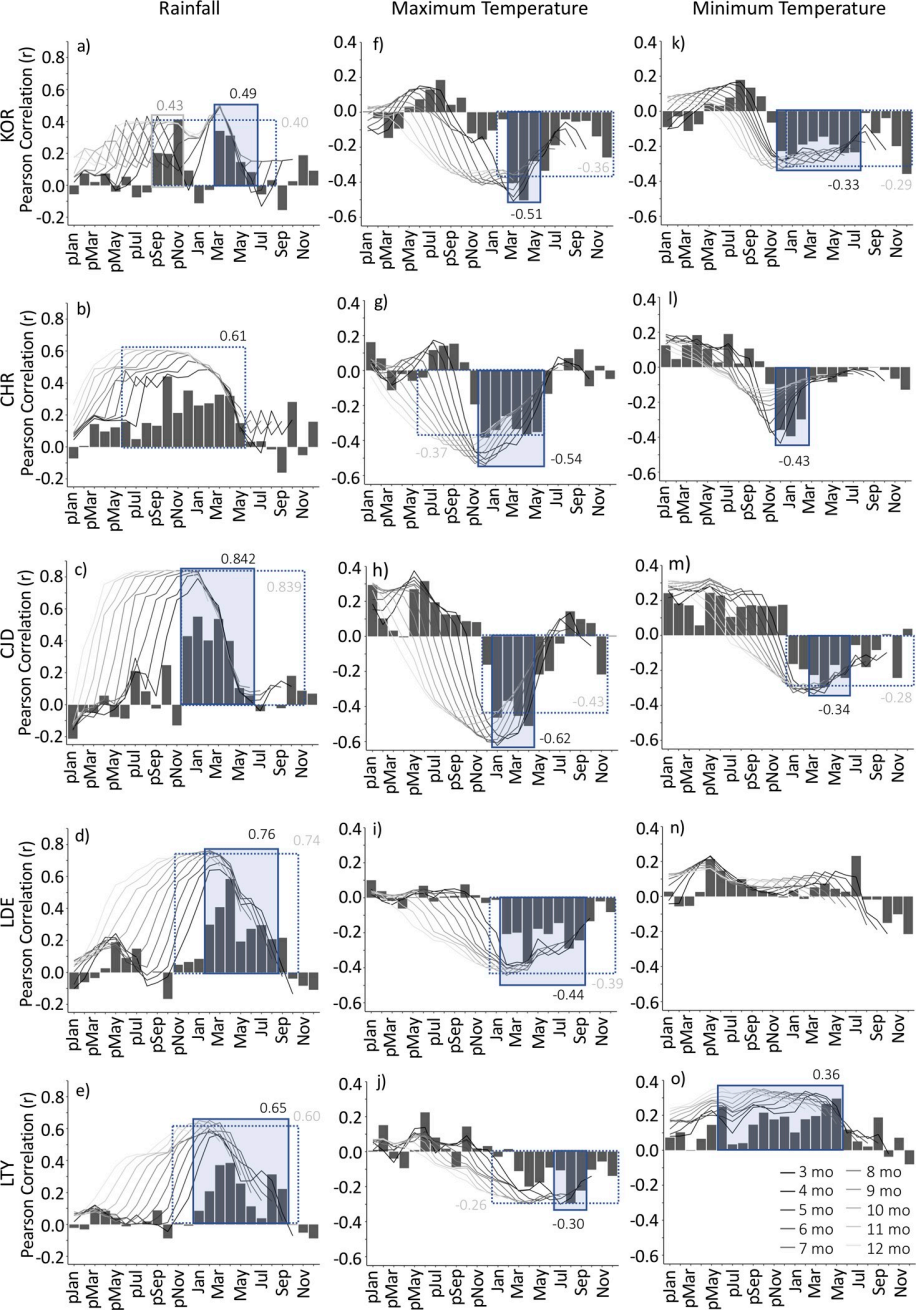
Pearson correlations between ring-width index (RWI) and monthly (columns) and seasonal (lines) rainfall (a-e), maximum (f-j) and minimum temperatures (h-o) at each of the five *Callitris columellaris* sites. The x-axis indicates the starting month of the seasonal period while the colours of the lines correspond to the length of the seasonal period (the number of consecutive months) rainfall was summed over or temperature was averaged over. Shaded boxes indicate the ‘best’ (highest correlation) season; dashed boxes indicate the best annual period for each site. Numbers inside or next to boxes are the Pearson correlation values for the respective season or annual period. Lower case ‘p’ indicates months in the calendar year prior to the main growing season. LDE showed no significant correlation between RWI and minimum temperature for any period > 3 months.

Our five sites exhibit distinctly different seasonal climatic patterns, particularly in terms of the timing and duration of wet and dry seasons (S1 Fig). Consequently, we defined different annual and ‘growing season’ periods for each site based on correlations between seasonal and annual total rainfall and RWI. We used the *monthly_response()* function in the DendroTools package [46] in R 3.6.1 [47] to calculate Pearson correlations between RWI and total rainfall for each month as well as for all periods between 3 and 12 consecutive months out of a possible 24-month window (previous calendar year plus current calendar year). At all five sites, growth (RWI) was significantly correlated with monthly rainfall over several months as well as total annual rainfall (albeit in different 12-month periods for each site; Fig 2A–2E). For the semi-arid sites, RWI was most strongly correlated with rainfall in the typical wet season (autumn-winter for the Mediterranean LTY and LDE sites, summer for the sub-tropical CJD site; Fig 2C–2E). At the tropical CHR site, rainfall in each of the typical wet season months (Oct-Apr) showed significant correlations with RWI; however, RWI was most strongly related to rainfall over a 12-month period from the previous dry season to the end of the typical wet season (pJun to May; r = 0.614; Fig 2B) rather than to the wet season months alone. Rainfall summed over shorter periods between 7–11 months also showed similarly strong correlations with RWI (r < 0.61, Δ R^2^ < 0.02), but nevertheless, slightly weaker than the best annual period (Fig 2B). At the wet tropical site, KOR, growth was significantly related to rainfall in the months prior to (Sep-Nov) and following (Mar-Jun) the main wet season, but not related to rainfall in the wettest months of the wet season (pDec-Feb; Fig 2A). Rainfall in the 4-month Mar-May period i.e. post wet season, showed the strongest relationship with RWI out of any of the periods we tested (Fig 2A). The annual and ‘growing season’ periods we selected for use in further analyses are summarised in Table 1.

Tree growth is also significantly correlated with temperature (Fig 2F–2O), but these relationships are weaker than those with rainfall (Fig 2A–2E) and are in large part the result of strong correlations between rainfall and temperature (i.e., periods of high rainfall are typically associated with a reduction in the diurnal temperature range; [48]) rather than direct effects of temperature on growth.

### Rainfall variables and regression models

We calculated total rainfall for annual and growing season periods for each site to use as variables to predict tree growth (as RWI) in regression models. For each of these rainfall amount variables, we fit both a simple linear model and a quadratic polynomial model with RWI as the response variable and the rainfall variable as the predictor, using the *lm()* function in the base package of R [47]. We used F-tests (the *anova()* function in R) to test if a polynomial model fit the data significantly better than a simple linear model. We then compared the goodness of fit among the different models, selecting the ‘best’ model(s) as the one(s) with the highest R^2^. If other models with the same number of parameters had an R^2^ within 0.02 of the best model (Δ R^2^ < 0.02), we considered these to be equally supported as the ‘best’ models. For these data, this Δ R^2^ threshold is more conservative than the generally accepted criterion of Δ AIC > 2.

In order to determine whether other attributes of the rainfall distribution influence tree growth, we also calculated a range of annual rainfall variables from daily rainfall data that represent the intensity, frequency and intermittency of rainfall from all rain days (Table 2). We used simple linear models to determine if there were significant relationships between RWI and each of these rainfall variables.

**Table 2:** Rainfall variables used to examine the relationship between growth of *Callitris columellaris* and rainfall amount, intensity, frequency, and intermittency.

Variable	Definition	Unit
Amount of rain	Total amount of rainfall	mm
Rain intensity	Total amount of rainfall divided by the number of days with rainfall > 0mm	mm
Number of rain days	Number of days with rainfall > 0 mm	Days
Wet season length	Number of days between when 10% and 90% of the total annual rainfall was recorded	Days
Max consecutive dry days	Maximum number of consecutive days without rainfall (0 mm)	Days
Max consecutive rain days	Maximum number of consecutive days with rainfall (>0 mm)	Days
Mean consecutive dry days	Mean number of consecutive days without rainfall (0 mm)	Days
Mean consecutive rain days	Mean number of consecutive days with rainfall (>0 mm)	Days

## Results

### Sensitivity of growth to inter-annual variation in the amount of rainfall

**Fig 3:**
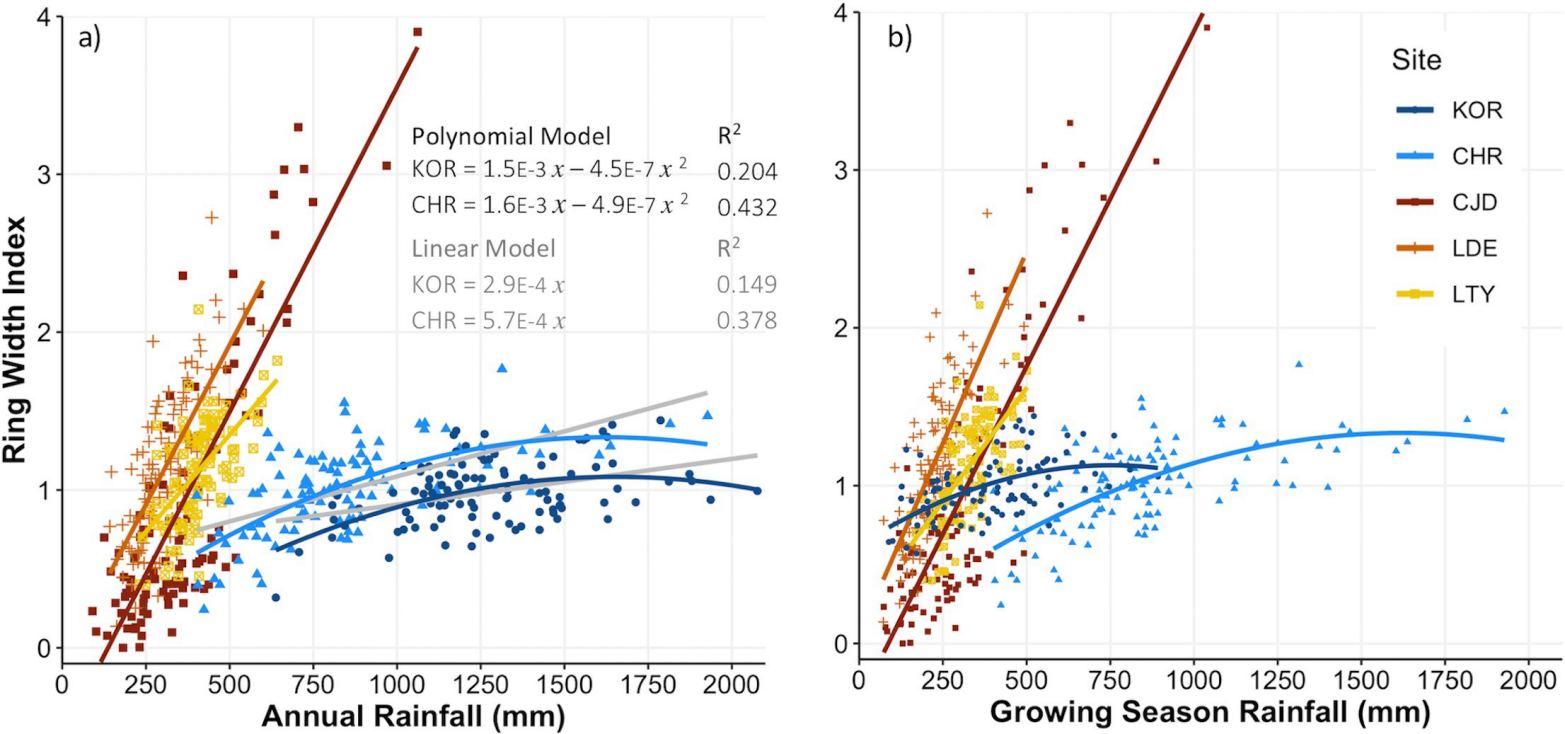
Relationship between ring-width index (RWI) and (a) annual or (b) growing season rainfall amount at the five *Callitris columellaris* sites. Each point represents data for one year. Lines represent fitted models; simple linear models for CJD, LDE and LTY and quadratic polynomial models for KOR and CHR. The fit of simple linear models for KOR and CHR are also shown in grey in (a).

**Fig 4:**
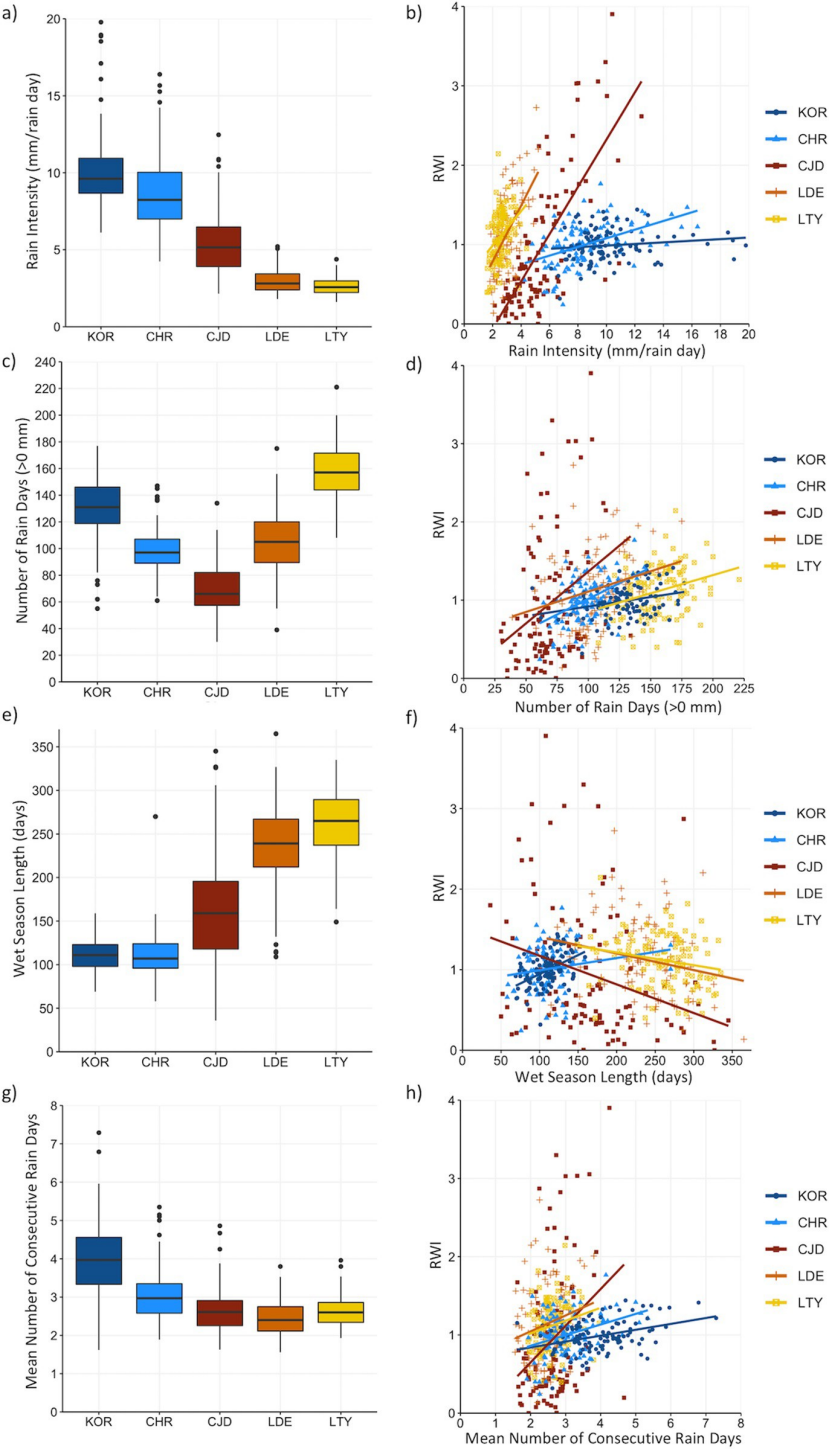
Distribution of annual rainfall attributes and their relationship with Ring Width Index (RWI) of *Callitris columellaris* across semi-arid (yellow-red) and tropical (blue) biomes in Australia. (a-b) mean rain intensity (mm/rain day), (c-d) number of rain days (>0 mm), (e-f) length of wet season (number of days) and (g-h) mean number of consecutive rain days (>0 mm). Boxplots show the median (50th percentile) as the centre horizontal line, the interquartile range (25th to 75th percentile) as the bottom and top horizontal line, the range (0.3–99.7th percentile) as vertical lines, and extreme values (<0.7 or >99.3 percentile) of mean monthly temperatures as dots. Lines in b), d), f), and h) represent simple linear models.

At all sites, tree growth (as RWI) was significantly and positively related to rainfall amount (Fig 3) but the strength and shape of the relationship varied among semi-arid and tropical biomes. In semi-arid biomes, growth was strongly and linearly related to rainfall amount (Fig 3 and Table 3). The slopes of the linear models were greatest at the LDE and CJD sites (slope > 0.0041*x* for both annual and growing season rainfall; Table 3), despite differences in rainfall seasonality (S1 Fig), frequency and intensity (Fig 4A and 4C) between these two sites. The LTY site also showed a strong growth response to annual or growing season rainfall amount, albeit slightly weaker (lower slope and lower model R^2^; Fig 3 and Table 3A and 3B) than the other two semi-arid sites.

**Table 3:** Parameter estimates for regression models predicting ring-width index (RWI) from a) annual rainfall amount and b) “growing season” rainfall amount.

**a)**									
**Site**	**Int.**	**a**		**SE**	**b**		**SE**	**Model R**^**2**^	**DF**
KOR	-0.15	1.48E-03	**	4.56E-04	-4.52E-07	**	1.69E-07	0.204	102
CHR	0.04	1.60E-03	***	3.30E-04	-4.91E-07	**	1.55E-07	0.432	106
CJD	-0.56	4.12E-03	***	2.63E-04				0.704	103
LDE	-0.10	4.14E-03	***	3.63E-04				0.536	106
LTY	0.12	2.46E-03	***	3.19E-04				0.361	105
**b)**									
KOR	0.62	1.34E-03	**	4.07E-04	-8.93E-07	.	4.64E-07	0.266	102
CHR	0.04	1.60E-03	***	3.30E-04	-4.91E-07	**	1.55E-07	0.432	106
CJD	-0.37	4.25E-03	***	2.68E-04				0.709	103
LDE	0.05	4.85E-03	***	3.97E-04				0.583	106
LTY	0.18	2.88E-03	***	3.28E-04				0.423	105

Note: CJD, LDE and LTY were fitted with simple linear models (i.e., *y* = intercept + a*x*); KOR and CHR were fitted with quadratic polynomial models (i.e., *y* = intercept + a*x* + b*x*^2^). SE = standard error of the parameter estimate. Asterisks/NS indicates whether the parameter estimate is significantly different from zero:

*** *p* < 0.001;

** *p* < 0.01;

* *p* < 0.01;. *p* < 0.1, NS = not significant, *p* > 0.1. DF = Degrees of Freedom. See Table 1 for definitions of growing seasons for each site.

In contrast, the relationship between growth and rainfall amount in the tropics was better described by a concave-down polynomial model than a simple linear model (p < 0.05; Fig 3). Growth in the tropical biomes also showed a smaller response (lower slope) to inter-annual variation in rainfall amount than in the semi-arid biomes (Fig 3 and Table 3). The concave-down shape of the polynomial model in the tropical biomes indicates that growth is considerably less responsive (lower slope) to variation in rainfall at the higher end (> ~1,250 mm annual rainfall) than at the lower end (< ~1,000 mm annual rainfall) of the rainfall range.

### Sensitivity of growth to inter-annual variation in the frequency, duration and intensity of rainfall

Growth (RWI) was positively related to rain intensity across all biomes, with the exception of the wet-tropical KOR site, where rain intensity was the highest of the five sites (Table 4 and Fig 4A and 4B). The number of rain days (>0 mm) was a significant and positive predictor of growth across all biomes, but explained very little of the variation in growth (less than 10%), except at the tropical CHR site, where it explained ~ 23% of the variance in growth (Table 4 and Fig 4D). The semi-arid sites showed weak negative relationships with wet season length, while the tropical sites showed positive relationships with wet season length (Fig 4F). Only the wet-tropical KOR site showed a relatively strong relationship with wet season length, where it explained more than 20% of the variation in growth, as much as annual rainfall (Table 4 and Fig 4F). The number of consecutive wet days (mean or maximum) was a better predictor of growth than the number of consecutive dry days, with growth at all sites showing positive relationships with the mean or maximum number of consecutive wet days. However, the mean or maximum number of consecutive wet days were only weak predictors of growth, explaining <14% of variance in RWI across all sites (Table 4 and Fig 4H).

**Table 4:** Slope parameter estimates and variance in ring-width index (RWI) explained by annual rainfall variables.

	Tropical						Sub-tropical semi-arid			Mediterranean semi-arid					
	KOR			CHR			CJD			LDE			LTY		
	R^2^	Slope	*p*	R^2^	Slope	*p*	R^2^	Slope	*p*	R^2^	Slope	*p*	R^2^	Slope	*p*
Rain intensity	0.018	0.010	^NS^	0.279	0.546	***	0.535	0.298	***	0.345	0.350	***	0.130	0.218	***
Number of rain days	0.077	0.002	**	0.234	0.008	***	0.092	0.013	**	0.067	0.005	**	0.087	0.005	**
Wet season length	0.208	0.005	***	0.028	0.002	^.^	0.074	-0.004	**	0.049	-0.002	*	0.042	-0.002	*
Mean wet days	0.133	0.075	***	0.118	0.136	***	0.098	0.496	**	0.040	0.204	*	0.040	0.165	*
Max wet days	0.027	0.003	^.^	0.044	0.008	*	0.049	0.050	*	0.072	0.034	**	0.014	0.010	^NS^
Mean dry days	0.000	0.000	^NS^	0.050	-0.041	*	0.029	-0.044	^.^	0.037	-0.068	*	0.074	-0.182	**
Max dry days	0.000	0.001	^NS^	0.037	-0.002	*	0.000	0.000	^NS^	0.005	-0.002	^NS^	0.003	-0.003	^NS^

Note: All models were simple linear regression models. Asterisks indicate significance of the variable as a predictor in a linear regression model (i.e., whether the slope parameter estimate is significantly different from zero):

*** *p* < 0.001;

** *p* < 0.01;

* *p* < 0.05;. = *p* < 0.1; NS = not significant, *p* > 0.1. See Table 2 for definitions of each variable.

## Discussion

Our study used tree-ring records to provide a century-long perspective on how temporal variation in the amount, intensity and timing of rainfall impacts the growth response of trees and how these relationships differ among biomes. We found that the sensitivity of growth to changes in rainfall amount differs substantially between semi-arid and tropical biomes of Australia and is the highest (greatest slope) in semi-arid biomes. This finding is consistent with observations that the sensitivity of primary production to year-to-year changes in rainfall decreases along continental-scale gradients of increasing mean annual rainfall (e.g., [5, 6, 21, 49]). We also found that the shape of the temporal relationship between growth and rainfall amount differs between semi-arid and tropical biomes of Australia (Fig 3), which has important implications for estimating how growth rates are likely to respond to potential changes in rainfall amount or variability.

Growth of *C*. *columellaris* in semi-arid Australia is strongly and symmetrically (linearly) related to rainfall amount; i.e., growth responses to both wet and dry years are of a proportional magnitude. This linear relationship holds across the 10-fold range of rainfall observed in the last century (< 100 to > 1000 mm annual rainfall; Fig 3), indicating that water availability is the primary limitation to growth of *C*. *columellaris* in semi-arid biomes even during ‘wet’ years and that other factors such as solar radiation or nutrient availability are unlikely to significantly limit growth during periods of high water availability. This finding is in contrast to observations of negative asymmetric (i.e., concave down) growth responses to cool-season rainfall amount in trees in the semi-arid west of the United States [17]. However, our results are more broadly in agreement with and provide some support for recent studies that concluded that positive asymmetry dominates the carbon cycle in semi-arid Australia [50, 51], where dramatic increases in primary productivity and carbon uptake occur during extremely high rainfall years [50, 52]. While our results show a symmetrical (linear) rather than a positive asymmetrical relationship between tree growth and rainfall amount in semi-arid biomes of Australia, the slope of the linear response is steep (particularly at the CJD and LDE sites) and shows large increases in growth during years of extremely high rainfall. We suggest our results, along with those of previous studies [50–52] indicate that both short-term productivity responses and longer-term growth patterns in semi-arid Australia are highly sensitive to rainfall extremes.

The steep and symmetric (linear) relationship between tree growth and rainfall amount in semi-arid Australia suggests that changes in the mean amount of rainfall are likely to have significant effects on mean growth rates; a decline in mean rainfall would result in a proportional decline in mean growth rates and vice versa [5–7]. For semi-arid southern Australia, our findings thus indicate that a projected continuation of a recent multi-decadal declining trend in winter rainfall in the coming decades [53] is likely to result in significant declines in tree growth rates and thus ecosystem carbon sequestration (e.g., [54]). For the semi-arid northern and arid interior of Australia projected changes in mean rainfall are less certain, but there is high confidence that the intensity of extreme rainfall events and the time spent in drought will increase [53]. If such changes lead to greater inter-annual variability of rainfall without a change in the mean amount of rainfall, our findings indicate that inter-annual variability of tree growth will likely increase but long-term mean growth rates will not change because growth responses to both extreme wet and extreme dry years are expected to be of a proportional magnitude [5–7].

In contrast to semi-arid biomes, tree growth in the Australian tropics showed a negative asymmetrical (concave down or ‘saturating’) response to rainfall amount (Fig 3), which is consistent with the concept that productivity in mesic biomes is less sensitive to variation in rainfall amount at the wet end of the annual rainfall range than at the dry end [5, 6, 13, 14]. Productivity and growth are likely less responsive to rainfall amount in wet years in mesic biomes because much of the rainfall in high rainfall years may be lost to runoff [55, 56] or because other resources (i.e., light and/or nutrients) may become limiting to productivity and growth when water availability is high [57–59]. Similar explanations are likely behind our finding at the wet-tropical KOR site that tree growth was not related to rainfall amount during the core wet season months (Dec-Feb; Fig 2A; a finding that was also noted by Allen et al. [31]), which typically receive >200 mm/month (S1 Fig). Grass and tree productivity in tropical northern Australia have also been shown to be limited during the core wet season (i.e., the most active period of the summer monsoon, Dec-Mar) when soil water conditions are conducive to growth, but thick cloud cover limits the capacity of vegetation to absorb solar radiation [58, 60]. Hence, in contrast to semi-arid biomes, our findings suggest that water availability in tropical Australia is not consistently the primary limiting factor for tree growth; instead other factors likely become limiting to growth when water availability is high.

The sensitivity of growth to inter-annual variation in rainfall amount is weakest in the most mesic biome we examined (i.e., the wet-tropical KOR site had the lowest R^2^ and slope); however, the variance explained (R^2^) is within the range that has been found for trees growing in mesic biomes elsewhere (>1,700 mm; e.g., [61–63]). The relatively weak R^2^ at KOR may be partially explained by our selection of a short 4-month ‘growing season’ Mar-Jun), which potentially doesn’t fully capture the rainfall period that is most important to growth in the wet tropics; that is, rainfall in the transitional months both before (Sep-Nov) and after (Mar-Jun) the main wet season (Fig 2A; see also [31]). Including the pre-wet season months (Sep-Nov) in ‘growing season’ rainfall totals for KOR does improve the variance in RWI explained by rainfall amount (by 8%), but does not significantly alter the negative-asymmetric shape or slope of the relationship between tree growth and rainfall amount (S4 Fig). In addition, our finding, along with others [24, 31, 64, 65] that the seasonal timing, duration and frequency of rainfall are as important (perhaps more so) for driving growth of *C*. *columellaris* as the total amount of rainfall in the wet tropics of Australia (>1,200 mm annual rainfall) may also partially explain the relatively weak relationship between growth and rainfall amount.

While there is large uncertainty around potential future changes in mean rainfall for tropical northern Australia [53], the relatively flat and negative asymmetrical response of growth to variation in rainfall amount in tropical biomes of Australia suggests that tree growth rates are relatively insensitive to potential changes in mean rainfall, particularly if mean rainfall was to increase [5–7]. However, if inter-annual variability of rainfall increases (i.e., an increase in the frequency and/or severity of extreme wet or dry years) and mean rainfall remains unchanged, mean growth rates will likely decrease in relation to the present mean, because declines in growth in response to dry years are expected to be larger than increases in growth in response to wet years [5–7]. In addition, the significant influence of wet season length, duration of wet periods (mean number of consecutive wet days; Table 4), and the seasonal timing of rainfall in the tropics (Fig 2A) suggests that changes in the intra-annual distribution of rainfall are likely to also have significant impacts on growth in the tropics. For example, if annual rainfall in tropical Australia is redistributed so that wet seasons become wetter and dry seasons become drier, as has been observed in parts of the Australian tropics over the last century (i.e., 1930–1990 CE, [8]; 1950–2009 CE, [57]), productivity and growth rates would be expected to decline, because a greater proportion of rainfall would be lost to runoff [56, 57, 66].

In this study, we have used an “indicator” species (*Callitris columellaris*) growing across a latitudinal gradient that also encapsulates a gradient in hydroclimatic stress to reveal potential growth responses to changing rainfall patterns across mesic and water-limited biomes of Australia. We expect our findings are more broadly applicable to other shallow-rooted woody species; however, additional research efforts are needed both in Australia and elsewhere to better understand how the long-term sensitivity of tree growth to rainfall variability and amount varies among species and how, for example, this relates to observations of asymmetry made at much larger spatial scales.

## Supporting information

S1 Fig Monthly (a-e) total rainfall and (f-j) monthly mean minimum and maximum temperatures at the five Callitris columellaris sites. Boxplots show the median (50th percentile) as the centre horizontal line, the interquartile range (25th to 75th percentile) as the bottom and top horizontal line, the range (0.3–99.7th percentile) as vertical lines, and extreme values (<0.7 or >99.3 percentile) of mean monthly temperatures as dots. Numbers inside or next to boxes in f-o are the Pearson correlation values for the respective season or annual period. LDE showed no significant correlation between RWI and minimum temperature for any period > 3 months. Rainfall data are from Australian Bureau of Meteorology stations (see S1 Table). Temperature data are from the SILO database (https://www.longpaddock.qld.gov.au/silo/), downloaded for the nearest 0.5° grid point to each site.
(TIFF)

S2 Fig a) Total annual rainfall and b) annual raw ring width (sample mean) of the five Callitris columellaris sites. Boxplots show the median (50th percentile) as the centre horizontal line, the interquartile range (25th to 75th percentile) as the bottom and top horizontal line, the range (0.3–99.7th percentile) as vertical lines, extreme values (<0.7 or >99.3 percentile) and the mean (diamonds) of annual total rainfall and ring width. Note: Raw ring widths are the sample mean ring width measurement (in mm) for each year. Raw ring widths have not been detrended to remove age-related (non-climatic) growth trends and are shown here only to provide an indication of differences in actual ring widths (growth rates) among sites and rainfall zones (RWI is standardized to a mean of one across all sites).
(TIFF)

S3 Fig Measures of signal strength, the expressed population signal (EPS) and the RBAR, and the sample depth (n) of each of the a) LTY, b) LDE and c) CHR chronologies for the period > 1900 CE. RBAR provides an indication of chronology signal strength (common variance) and is independent of sample size [67]. The EPS provides an indication of the likely loss of reconstruction accuracy as a function of RBAR and sample size, measuring how well the finite-sample chronology compares with the theoretical population chronology based on an infinite number of trees [68].
(TIFF)

S4 Fig Relationships between tree growth (ring width index) and rainfall amount over different ‘growing seasons’ at the tropical KOR site. Each point represents data for one year. Lines represent fitted quadratic polynomial models.
(TIFF)

S1 Table Details for the Australian Bureau of Meteorology stations used to calculate rainfall variables for each of the *Callitris columellaris* sites. (DOCX)
