# Supplementary figures and images for: Tree growth responses to temporal variation in rainfall differ across a continental-scale climatic gradient
Source: PLoS One. 2021 May 4;16(5):e0249959. doi: 10.1371/journal.pone.0249959 (PMC8096069; doi:10.1371/journal.pone.0249959)

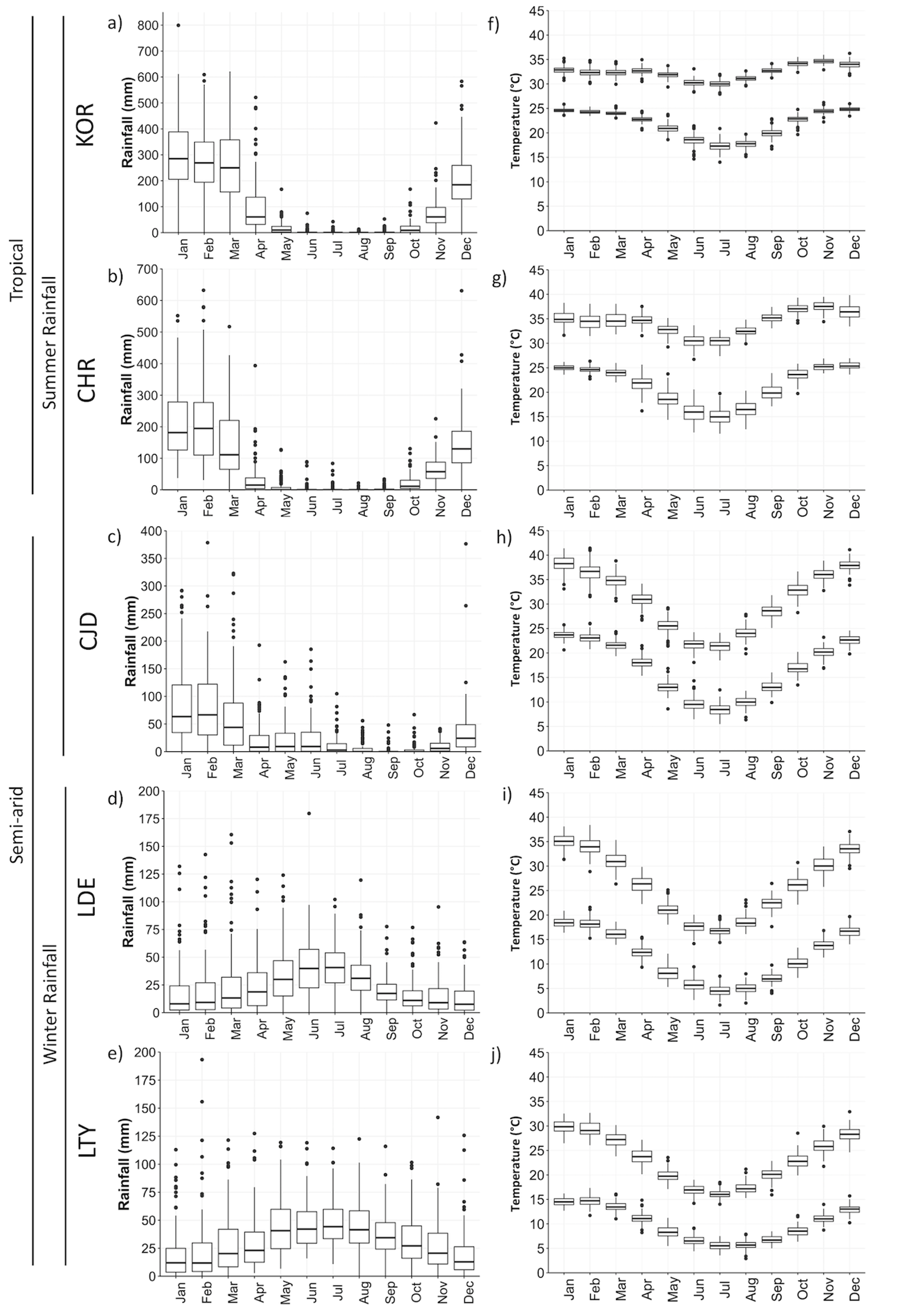

Supplement: S1 Fig — Monthly (a-e) total rainfall and (f-j) monthly mean minimum and maximum temperatures at the five Callitris columellaris sites. Boxplots show the median (50th percentile) as the centre horizontal line, the interquartile range (25th to 75th percentile) as the bottom and top horizontal line, the range (0.3–99.7th percentile) as vertical lines, and extreme values (<0.7 or >99.3 percentile) of mean monthly temperatures as dots. Numbers inside or next to boxes in f-o are the Pearson correlation values for the respective season or annual period. LDE showed no significant correlation between RWI and minimum temperature for any period > 3 months. Rainfall data are from Australian Bureau of Meteorology stations (see S1 Table). Temperature data are from the SILO database (https://www.longpaddock.qld.gov.au/silo/), downloaded for the nearest 0.5° grid point to each site. (TIFF) [file pone.0249959.s001.tiff]

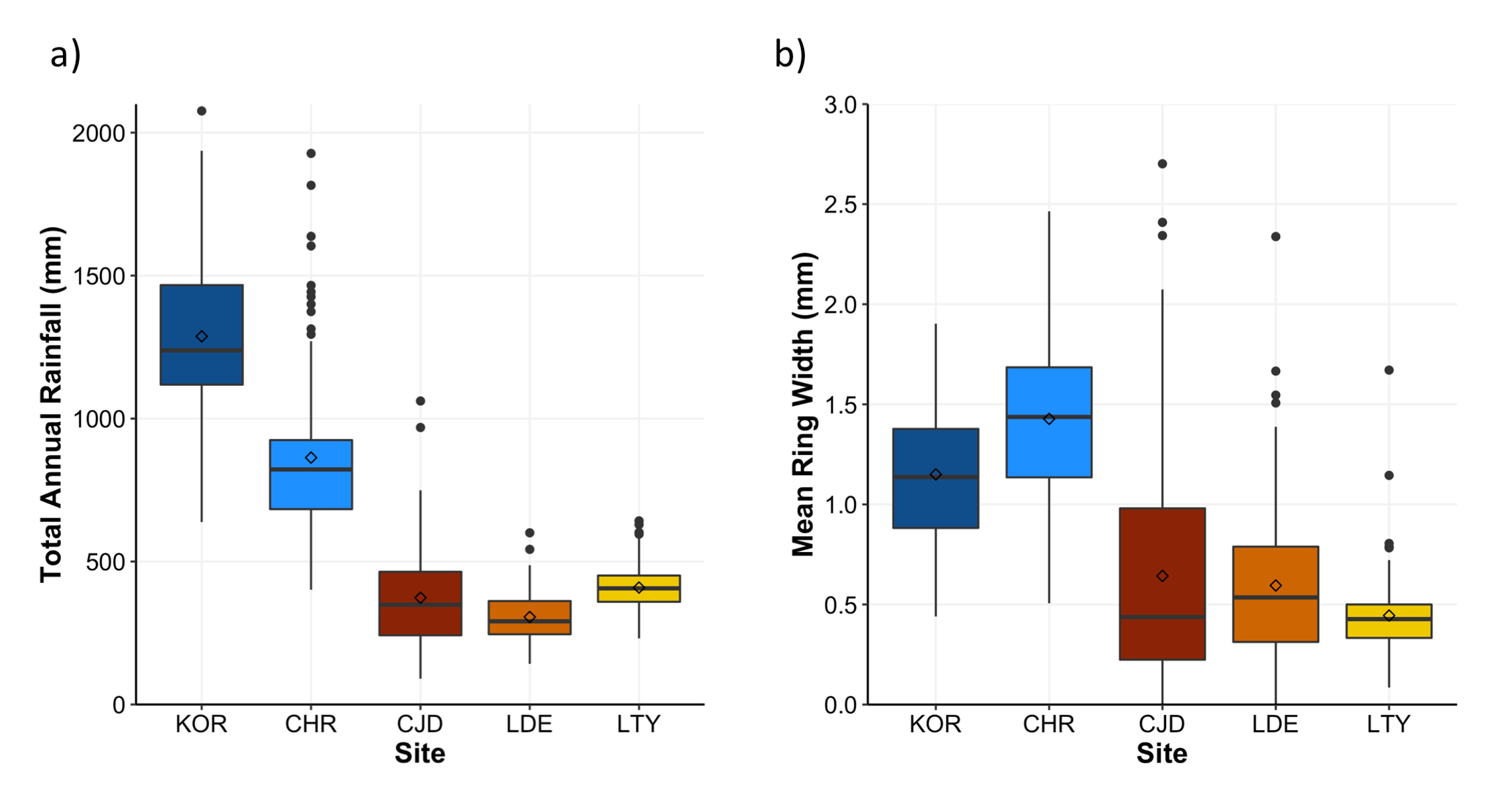

Supplement: S2 Fig — a) Total annual rainfall and b) annual raw ring width (sample mean) of the five Callitris columellaris sites. Boxplots show the median (50th percentile) as the centre horizontal line, the interquartile range (25th to 75th percentile) as the bottom and top horizontal line, the range (0.3–99.7th percentile) as vertical lines, extreme values (<0.7 or >99.3 percentile) and the mean (diamonds) of annual total rainfall and ring width. Note: Raw ring widths are the sample mean ring width measurement (in mm) for each year. Raw ring widths have not been detrended to remove age-related (non-climatic) growth trends and are shown here only to provide an indication of differences in actual ring widths (growth rates) among sites and rainfall zones (RWI is standardized to a mean of one across all sites). (TIFF) [file pone.0249959.s002.tiff]

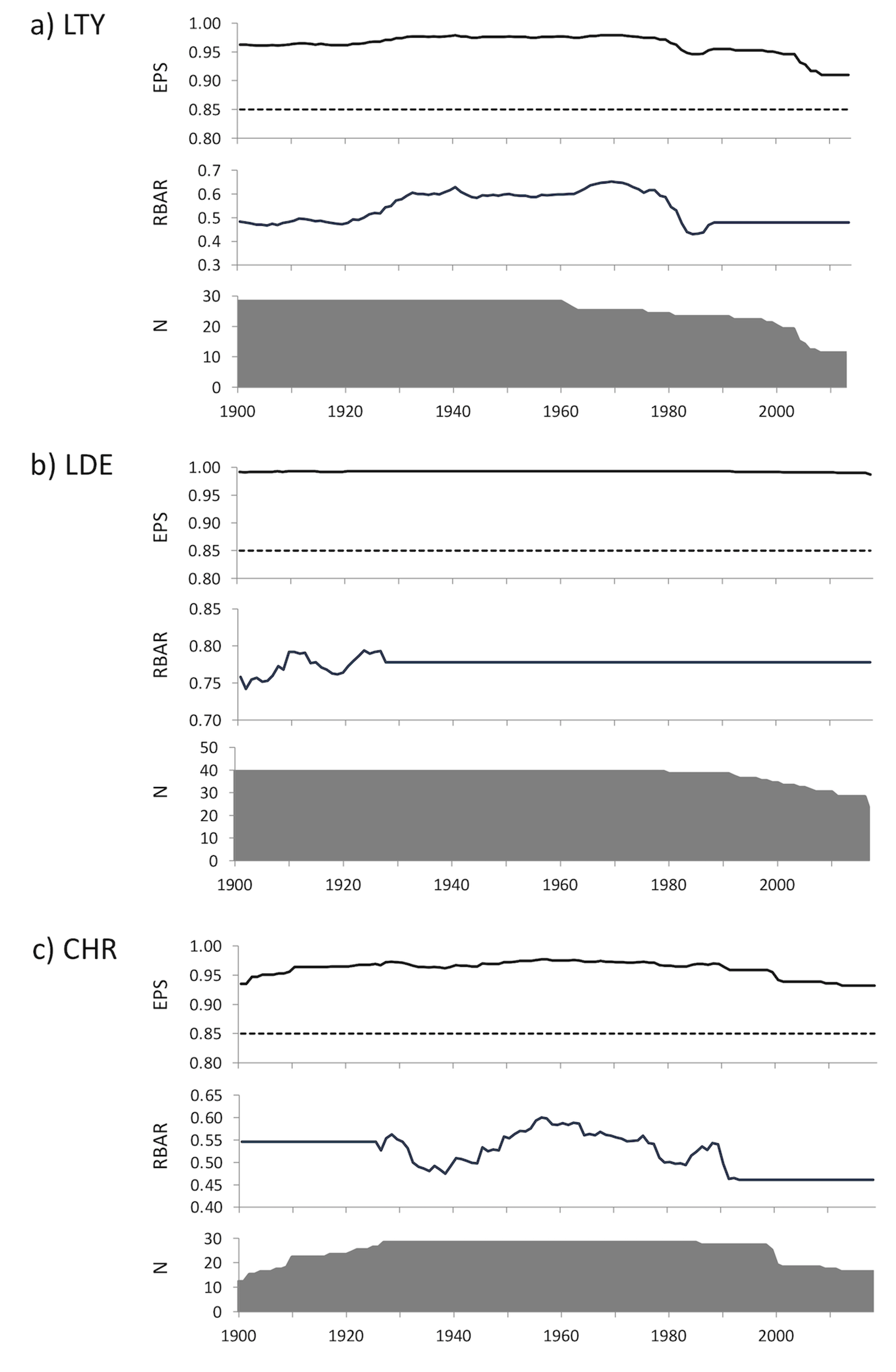

Supplement: S3 Fig — Measures of signal strength, the expressed population signal (EPS) and the RBAR, and the sample depth (n) of each of the a) LTY, b) LDE and c) CHR chronologies for the period > 1900 CE. RBAR provides an indication of chronology signal strength (common variance) and is independent of sample size [67]. The EPS provides an indication of the likely loss of reconstruction accuracy as a function of RBAR and sample size, measuring how well the finite-sample chronology compares with the theoretical population chronology based on an infinite number of trees [68]. (TIFF) [file pone.0249959.s003.tiff]

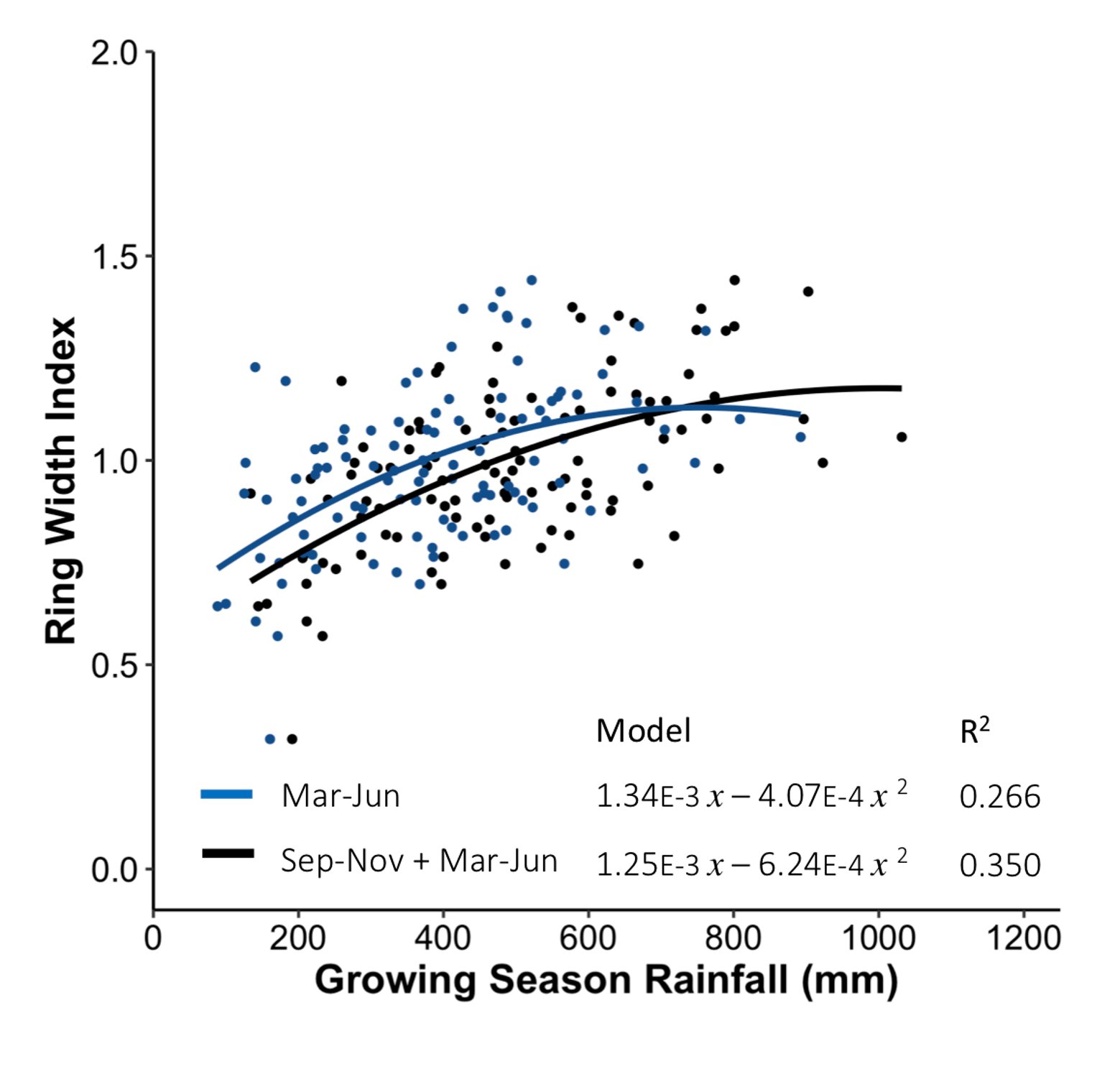

Supplement: S4 Fig — Each point represents data for one year. Lines represent fitted quadratic polynomial models. (TIFF) [file pone.0249959.s004.tiff]
