# Supplementary material for: Tree growth responses to temporal variation in rainfall differ across a continental-scale climatic gradient
Source: PLoS One. 2021 May 4;16(5):e0249959. doi: 10.1371/journal.pone.0249959 (PMC8096069; doi:10.1371/journal.pone.0249959)
Supplement: S1 Table — (DOCX) [file pone.0249959.s005.docx]

**S1 Table.** **Details for the Australian Bureau of Meteorology Stations used to calculate rainfall variables for each of the *Callitris columellaris* sites.**

|  | **Site Location** | |  | |  | |  | | **Station Location** | | |  | |  | |  | |
| --- | --- | --- | --- | --- | --- | --- | --- | --- | --- | --- | --- | --- | --- | --- | --- | --- | --- |
| **Site** | **Lat (°S)** | **Lon (°E)** | **Search Area** | **Name** | | **Years of Data** | | **Lat (°S)** | | **Lon (°E)** | **Dist**  **(km)** | | **Dir** | | **Comp (%)** | |  |
| KOR | 12.55 | 134.37 | 11.5-13.5°S 133-135°E | Oenpelli | | 1912-2013 | | 12.32 | | 133.06 | 144 | | NW | | 97 | |  |
|  |  |  |  | Warruwi | | 1916-2019 | | 11.65 | | 133.38 | 146 | | NW | | 93 | |  |
|  |  |  |  | Milingimbi | | 1923-2003 | | 12.12 | | 134.91 | 75 | | NE | | 82 | |  |
| CHR | 15.09 | 128.68 | 14-16°S 127.5-129.5°E | Oombulgurri | | 1914-2011 | | 15.18 | | 127.85 | 90 | | SW | | 54 | |  |
|  |  |  |  | Wyndham Port | | 1886-1995 | | 15.46 | | 128.1 | 74 | | W | | 98 | |  |
|  |  |  |  | Carlton Hill | | 1897-2018 | | 15.49 | | 128.53 | 47 | | S | | 83 | |  |
|  |  |  |  | Ivanhoe Station | | 1907-2018 | | 15.69 | | 128.68 | 66 | | S | | 74 | |  |
|  |  |  |  | Kimberley Research Station | | 1944-2010 | | 15.65 | | 128.71 | 63 | | S | | 92 | |  |
|  |  |  |  | Legune | | 1957-2019 | | 15.21 | | 129.45 | 83 | | SE | | 97 | |  |
| CJD | 22.83 | 118.62 | 21-23°S 117.5-119.5°E | Hillside Station | | 1917-2018 | | 21.72 | | 119.4 | 146 | | NE | | 77 | |  |
|  |  |  |  | Abydos Woodstock | | 1907-1997 | | 21.62 | | 118.96 | 138 | | NE | | 67 | |  |
|  |  |  |  | Coolawanyah | | 1923-2019 | | 21.8 | | 117.81 | 141 | | NW | | 86 | |  |
|  |  |  |  | Hamersley | | 1912-2015 | | 22.28 | | 117.68 | 114 | | NW | | 91 | |  |
|  |  |  |  | Marillana | | 1936-2019 | | 22.63 | | 119.41 | 83 | | NE | | 80 | |  |
|  |  |  |  | Mount Florance | | 1907-2019 | | 21.79 | | 117.86 | 139 | | NW | | 89 | |  |
|  |  |  |  | Mulga Downs | | 1907-2018 | | 22.1 | | 118.47 | 82 | | NW | | 89 | |  |
| LDE | 30.68 | 119.27 | 29.5-31.5°S 118.5-120.5°E | Boodarockin | | 1935-2019 | | 31.06 | | 118.87 | 59 | | SW | | 99 | |  |
|  |  |  |  | Bullfinch | | 1911-2019 | | 30.98 | | 119.07 | 39 | | SW | | 99 | |  |
|  |  |  |  | Marvel Loch | | 1922-2019 | | 31.47 | | 119.49 | 90 | | SE | | 94 | |  |
|  |  |  |  | Moorine Rock | | 1924-2004 | | 31.31 | | 119.13 | 71 | | SW | | 85 | |  |
|  |  |  |  | Noongaar | | 1927-2019 | | 31.33 | | 118.97 | 77 | | SW | | 96 | |  |
|  |  |  |  | Southern Cross | | 1907-2007 | | 31.23 | | 119.33 | 61 | | SE | | 86 | |  |
|  |  |  |  | Turkey Hill North | | 1929-2019 | | 31.04 | | 119.31 | 40 | | S | | 98 | |  |
|  |  |  |  | Warralakin | | 1925-2007 | | 31.03 | | 118.56 | 78 | | SW | | 95 | |  |
| LTY | 33.02 | 120.74 | 32-34°S 119.5-121.5°E | Hopetoun | | 1907-2019 | | 33.93 | | 120.09 | 118 | | SW | | 87 | |  |
|  |  |  |  | Kayballup | | 1914-1986 | | 33.78 | | 119.94 | 112 | | SW | | 98 | |  |
|  |  |  |  | Lake King | | 1929-2019 | | 33.08 | | 119.68 | 99 | | W | | 80 | |  |
|  |  |  |  | Lake Camm | | 1929-2019 | | 32.93 | | 119.58 | 109 | | W | | 96 | |  |
|  |  |  |  | Mount Madden | | 1932-2019 | | 33.28 | | 119.78 | 112 | | SW | | 97 | |  |
|  |  |  |  | Ravensthorpe | | 1907-2019 | | 33.58 | | 120.04 | 70 | | SW | | 95 | |  |

Note: Lat = Latitude, Long = Longitude, Comp = completeness – percentage of days with measurements, Dist = distance from site (km), Dir = Direction from site.
